# Interaction between Growth Regulators Controls In Vitro Shoot Multiplication in Paulownia and Selection of NaCl-Tolerant Variants

**DOI:** 10.3390/plants11040498

**Published:** 2022-02-11

**Authors:** Jehan Salem, Ahmed Hassanein, Deiaa A. El-Wakil, Naglaa Loutfy

**Affiliations:** 1 Central Laboratory of Genetic Engineering, Botany and Microbiology Department, Faculty of Science, Sohag University, Sohag 82524, Egypt; ahmed.hassanein@science.sohsg.edu.eg; 2 Biology Department, Faculty of Science, Jazan University, Jazan 82817, Saudi Arabia; de107@yahoo.com; 3 Plant Pathology Research Institute, Agricultural Research Center, Giza 12619, Egypt; 4 Botany and Microbiology Department, Faculty of Science, South Valley University, Qena 83523, Egypt; naglaa.hassan@sci.svu.edu.eg

**Keywords:** plant growth regulators, micropropagation, salinity, esterase isoenzyme, RAPD, ISSR, somaclonal variation, paulownia

## Abstract

The interaction between cytokinin, auxin and GA controlled in vitro shoot multiplication in paulownia was influenced by a medium water potential (Ψ) modulation, where it was modulated using different textures or strengths of MS medium, media of different types (MS, WPM, SH and B5) or NaCl incorporation. The interaction between 2 mg/L BAP and 0.1 mg/L NAA expressed the highest shoot number on each media type, but it was better with media of lower water potential (MS and WPM), and MS medium was the best. Ψ of full-strength semisolid MS medium expressed the highest shoot multiplication. The opposite was detected when Ψ of MS medium was changed using half- or double-strength MS. Ψ of full-strength MS medium in semisolid form resulted in a valuable interaction between 2 mg/L BAP, 0.1 mg/L NAA and 0.1 mg/L GA, leading to efficient shoot formation, and it was associated with an increase in internode length and decrease in stem diameter, which facilitated obtaining synseeds with a high ability to convert. High genetic variation was recorded under long-term culture (14 subcultures). Polymorphism using the ISSR technique was higher than that of RAPD. A further increase in polymorphism was detected when NaCl was used, where five salt-tolerant lines were selected. Some salt-tolerant-selected lines showed one or more amplification products of a specific molecular weight that did not appear in the control. For example, with OPA-07 and OPG-02 RAPD primers, all the salt-tolerant-selected lines showed the appearance of amplification fragments (610 bp and 300 bp, respectively) that were not detected in control.

## 1. Introduction

Urban and farm areas are expanding all over the world, leading to the loss of natural forest systems resulting in global warming and changes in the rainfall patterns. It is creating a critical need for managed forestation projects to satisfy a growing worldwide market, especially in North Africa and the Middle East. Paulownia belongs to the Scrophulariaceae family with 40 chromosomes (2n = 2x = 40) and provides an ideal solution to this problem. It is a fast-growing tree that reaches the harvesting stage in ten years, has good quality timber, requires minimal management and little investment, and tolerates a range of temperate conditions (−10 °C–+45 °C) [1]. Paulownia wood is suitable for many industries, including furniture production, aircraft construction, packing boxes, musical instrument, shipbuilding, plywood, paper, cabinet-making, coffins, molding and secondary metabolites, including flavonoids, with high antioxidant activities [2,3]. 

To establish tissue culture protocols for a plant species, exogenous growth regulators are used to evoke certain responses. Indole-3-acetic acid (IAA), a-naphthalene acetic acid (NAA), indole-3-butyric acid (IBA) are commonly used auxins to promote root initiation. Cytokinins such as kinetin (6-furfurylaminopurine), BAP (benzylaminopurine), 2iP (2-isopentenyl-adenine and zeatin (4-hydroxy-3-methyl but-2-enylaminopurine) are used to promote cell division and differentiation. In addition, GA is used to stimulate elongation of internode and attain normal development of in vitro obtained plantlets [4]. Plant hormones rarely act alone and commonly show synergistic, antagonistic and additive interactions [5]. For example, auxins and cytokinins interact synergistically to control cell division and antagonistically to regulate adventitious bud and lateral root formation [6]. In addition, auxin and gibberellin interact to control stem elongation [7].

Plants subjected to treatment or overexpression of GA biosynthesis formed longer leaves, but plants subjected to GA inhibitors showed reduced leaf size [8]. Under shade conditions, auxin and gibberellin content in plant leaves increased, but cytokinin decreased, leading to the inhibition of their size by controlling cell proliferation and enlargement [9]. Cell enlargement requires activation of the cell wall and cytoplasmic esterases [10], leading to cell wall elongation and metabolism [11]. 

The effects of the applied growth regulators were influenced by their concentration, genotype, the crosstalk with each other synergistically or antagonistically and water potential (Ψ) of the culture medium [12,13]. The water potential of the medium is proportional to the concentration of its chemical constituents [14,15]. 

The differences in stress tolerance among cultivars or plant species are difficult to evaluate in the field where soil-grown plants are exposed to biological and climatic variable conditions [16]. Consequently, the in vitro culture techniques are preferred for carrying out a quick salinity tolerance test under controlled conditions. To improve the salt tolerance of salt-sensitive plants, such as paulownia [17], researchers focused their efforts on the selection of salt-tolerant genotypes [18]. Tissue culture can be used to generate a wide range of genetic variations and selection of a mutant with elite agronomic traits such as salt or drought tolerance.

Until commercialization, the micropropagation products require specific management including extensive roots formation, acclimatization and successful transfer to the soil. Synseeds technology is considered an alternative method to traditional micropropagation where somatic embryos, shoot tips or axillary buds are encapsulated within a synthetic endosperm and are covered externally with a synseed coat to suit mechanical handling and planting [19,20].

Selection from micro-propagated plants without genetic proof is totally unsatisfactory, where the detected trait may be attributed to a temporal metabolic change that will disappear with the disappearance of the applied factor. Investigation of genetic stability is necessary to evaluate if the detected variant is due to genetic or epigenetic factors [18]. Polymerase chain reaction (PCR)-based techniques, such as random amplified polymorphic DNA (RAPD) and inter simple sequence repeats (ISSR), have been used to investigate the genetic stability of in vitro-regenerated shoot lines in several crop species [21,22,23]. 

Paulownia witch’s broom (PWB) results from infection with PWB phytoplasma decreases tree growth causing seedling and tree death. The use of chemicals is not recommended due to the difficulties in getting them into the phloem where the pathogen parasitizes. In paulownia, the application of tissue culture techniques was used to produce pathogen-resistant paulownia [24], multiply pathogen-free plant materials [25] and produce pathogen-free plants [26,27]. However, there is insufficient information that takes into account the interaction between the different growth regulators and the interaction between them and the other organic and inorganic components of the nutritional medium, which can be generally expressed by the so-called water potential (Ψ). Hence, the aim of this work was to obtain an effective protocol that takes into account these interferences. We desire to create a protocol that makes applications of tissue cultures to improve the ability of the paulownia tree to resist abiotic factors, such as salinity, and free of biotic factors, such as pathogens, more feasible.

## 2. Results

The induction of shoot formation on paulownia nodal segments (Table 1) appeared in ten days on basal MS medium, but the number of formed shoots was low (1.7 shoots/explant) even with a low concentration of BAP up to 1 mg/L (2 shoots/explant). The maximum shoot formation appeared on MS medium supplemented with 2 mg/L BAP for four weeks where 3.7 shoots/explant were formed. It was associated with an increase in shoot length, the number of nodes and leaves and fresh weight of shoot/cluster. Increased BAP concentrations of more than 2 mg/L reduced the shoot formation and the measured growth parameters.

To study how the interaction between the selected concentration of BAP (2 mg/L) and NAA was affected by the water potential of the growth medium, paulownia explants were subcultured in different media (Table 2) and one medium (MS) with a different texture (Table 3) and different strengths (Table 4). When paulownia shoots were subcultured under the influence of 2 mg/L BAP, different concentrations of NAA and different types of the nutrient media (MS, B5, SH and Wm), the medium containing 2 mg/L BAP in combination with 0.1 mg/L NAA was the best regardless of medium type. It is noticeable that the appearance of calluses on the base of the cultured explant was necessary to increase shoot number. For example, explants grown on the SH medium did not show callus formation, so they formed the lowest number of shoots. The water potential generated by the nutrient MS medium in the semisolid or solid form expressed better shoot multiplication than liquid medium (Table 3). Changing the water potential of the medium by changing the medium strength strongly affected the multiplication efficiency of the paulownia tree, where the increase or decrease in the components of the full strength MS medium led to a significant decrease in shoot multiplication and growth (Table 4).

Shoot multiplication of the paulownia tree was influenced by cytokinin type (Table 5). While three types of cytokinins were used at the same concentration (2 mg/L), the best results were obtained as a result of the interaction between 2 mg/L BAP and 0.1 mg/L NAA.

**Figure 1:**
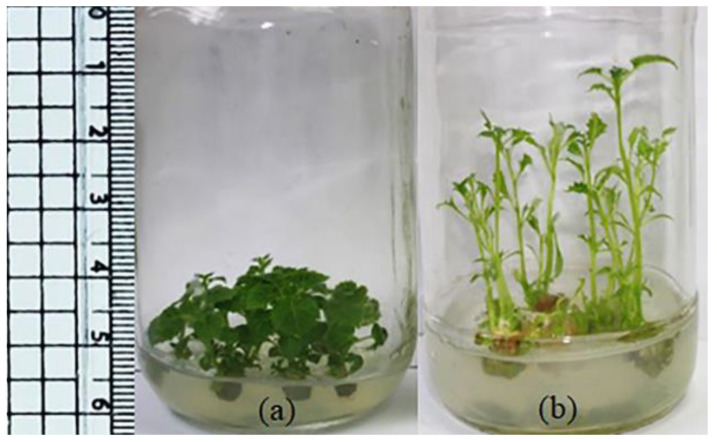
Multiplication of paulownia on semisolid MS medium containing 2 mg/L BAP and 0.1 mg/L NAA (**a**) without or (**b**) with 0.1 mg/L GA.

**Figure 2:**
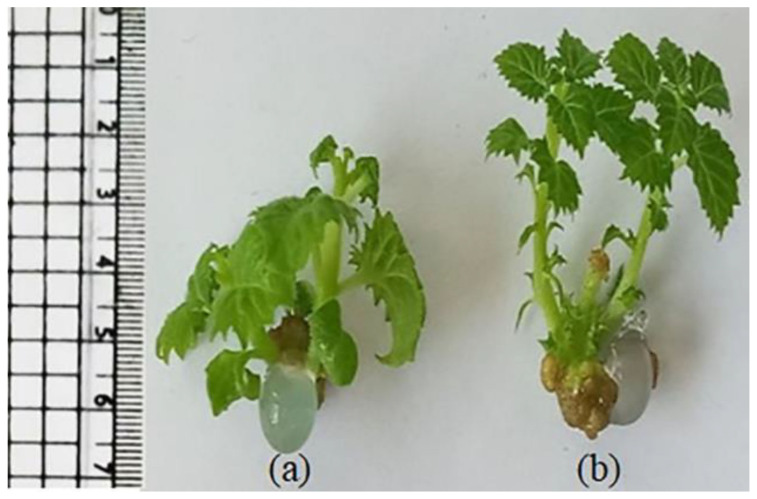
Conversion of synseeds (conserved for one month at 4 °C) on semisolid MS medium with 2 mg/L BAP and 0.1 mg/L NAA for one month. Encapsulated nodal explants were obtained from in vitro shoots grown on MS medium containing 2 mg/L BAP and 0.1 mg/L NAA (**a**) without or (**b**) with 0.1 mg/L GA.

The effect of interaction between BAP, NAA and GA on paulownia in vitro multiplication was investigated (Table 6). Replacing 0.1 mg/L of NAA with 0.1 mg/L GA in a medium containing 2 mg/L BAP reduced all measured values except for the length of shoot and internode. All the measured values increased when they were cultured on MS medium containing 2 mg/L BAP in combination with both 0.1 mg/L NAA and 0.1 mg/L GA_3_. Generally, the application of GA increased internode and shoot lengths but reduced leaf area and stem diameter (Table 6 and Figure 1). Application of high concentrations (3 or 4 mg/L) of BAP in combination with 0.1 mg/L NAA and 0.1 mg/L GA expressed valuable shoot multiplication (4.3 shoots/explant) but decreased the estimated growth values. The reduction in leaf area, increase in the length of internodes and decrease in stem diameter due to the incorporation of GA facilitated the formation of synseeds of paulownia and improved their conversion (Figure 2 and Table 7).

From the study of the esterase, isoenzyme patterns emerged that the plants that were multiplied on MS medium containing only GA differed from the plants that grew on other media (Figure S1). The density of bands of plants exposed only to GA was lower than that of the other plants that grew under the influence of a combination of more than one growth regulator. In addition, one band appeared (EST-8), but two bands (EST-1 and EST-6) disappeared. Plants were grown on a nutrient medium containing 2 mg/L BAP and 0.1 mg/L GA and were characterized by the appearance of two new bands (EST-1 and EST-6) regardless of the presence or absence of 0.1 mg/L NAA.

Induction of root formation was investigated under the impact of different concentrations of IBA, which was frequently used for in vitro root formation. The best data of root formation was obtained when full-strength MS medium supplemented with 1 mg/L IBA was used (Table 8). Further improvement in root formation was obtained when auxins were added in half-strength MS medium. Under these conditions, the stimulation of root formation by adding 1 mg/L NAA was better than 1 mg/L IBA, and the best results were obtained by using 0.5 mg of each. The lowest root number and length were obtained upon the application IAA (Table 8)

**Figure 3:**
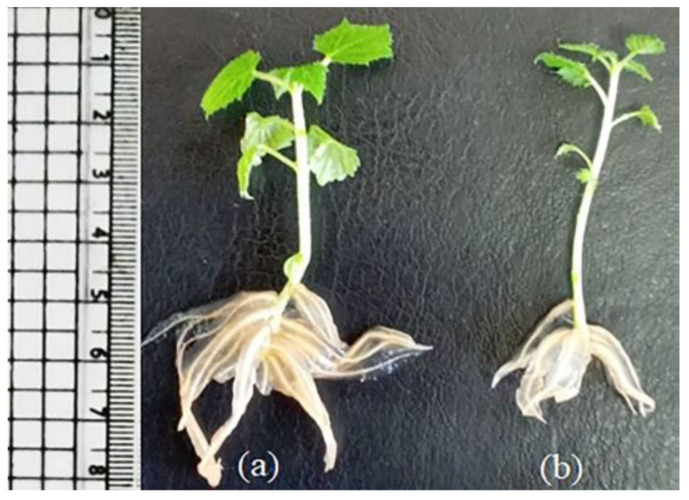
In vitro root formation on microshoots that were grown on MS medium containing 2 mg/L BAP and 0.1 mg/L NAA (**a**) without or (**b**) with 0.1 mg/L GA and were cultured for three weeks on semisolid half-strength MS medium with 0.5 mg/L IBA and 0.5 mg/L NAA.

The stimulated formation of roots on media containing 0.5 mg/L IBA and 0.5 mg/L NAA (root formation medium) was influenced by the presence or absence of 0.1 mg/L GA in the multiplication medium [MS with 2 mg/L BAP and 0.1 mg/L NAA (Table 9)]. Shoot cuttings that were transferred from the multiplication medium without GA to root formation medium were better than others that were transferred from the multiplication medium with GA, where under the influence of the last condition, the number of roots and their root growth was significantly reduced (Figure 3). The negative effect of GA on the establishment of an extensive root system and the decrease in stem diameter and leaf area led to a decrease in the percentage of plants that passed the acclimatization stage. Hence, the percentage of plants successfully transferred to the natural environment decreased from 80% in the absence of GA in multiplication medium to 28% in its presence, where all of them showed continuous growth upon their transfer to the open conditions (Figure S2).

The long-term culture was used as a tool to provoke somaclonal variation, as these plants were subjected to 14 subcultures one month each on a medium containing 2 mg/L BAP, 0.1 mg/L NAA and 0.1 mg/L GA. In addition, long-term culture in combination with NaCl in the same medium was used to select five NaCl-tolerant shoot lines. The selected lines tolerated the shock upon their transfer from salinity-free medium (12th and 13th subcultures) to medium with a sublethal concentration of NaCl, where the ability to multiply and grow came out, while all the plants that had not been treated before with salinity died.

Genomic DNA of ten different shoot lines of paulownia subjected to 14 subcultures was amplified using ten RAPD primers to detect somaclonal variation after long-term culture. The size of the amplified products ranged from 100–1300 bp (Table 10 and Figure S3). The highest number of bands (12) was scored by primer OPA-07, while the lowest number (3) by primer OPA-08. Out of 72 fragments, 29 were polymorphic bands (40.3%), where unique ones were considered polymorphic, but 43 bands were monomorphic (59.7%). Polymorphism ranged from 18.2% (primer OPA-02) to 80% (primer OPH-16; Figure S3), with a mean of 49.1%. The highest number of polymorphic bands (5) was detected when primers OPG-02 and OPR-11 were used and the lowest (1) with primer OPA-03 (Table 10).

When ten ISSR primers were used, the size of the amplified fragments ranged from 200 to 1000 bp (Table 10 and Figure S4). The highest number of bands (9) was obtained by primer UBC811, while the lowest number (2) by primers ISSR-5, ISSR-14 and 844 A. Twenty-five of all 51 bands were registered as polymorphic (49.02%), while 26 bands (50.98%) were monomorphic. The highest number of polymorphic bands (7) with a polymorphism of 100% was produced with primer ISSR-1 (Figure S4), but the lowest number (0) was obtained with primers ISSR-5, ISSR-14 and 844 A.

When the genomes of five salinity-tolerant selected shoot lines and that of one line were not treated with NaCl (control) were amplified using the same previous ten RAPD primers, 50 discrete bands ranging from 100 to 1500 bp in size were detected (Table 11). Of the 50 fragments scored, 23 were polymorphic (46%), and 27 were monomorphic (54%). The highest number of bands (10) was generated by primer OPA-02 and the lowest (2) by primers OPA-06, OPA-08 and OPH-16. The highest polymorphism (90%) was registered with primer OPA-02. The primers OPA-07 and OPG-02 distinguished among all the five lines that were selected as salinity-tolerant lines and the control, where new bands with molecular weights of 610 and 300 bp appeared, respectively (Figure S5). In addition, some salt-tolerant-selected lines showed one or more bands that were not detected in control, such as primer OPG-02 (lane 4) and OPBC-13 (lanes 4 and 5; (Figure S5)).

The previous ten ISSR primers were used to amplify the DNA genome of salinity-tolerant selected shoot lines and that of control, and 72 scorable bands were scored (Table 11), 37 of them were polymorphic (51.4%), but 35 were monomorphic (48.6%). The size of the amplification product ranged from 180 to 1200 bp. The highest number (10) of scorable bands was screened by the primer ISSR-3, while the lowest number (4) was by primers ISSR-2 and ISSR-5. In addition, primer ISSR-3 generated the highest number (6) of polymorphic bands, but UBC808 produced the lowest number (2). These polymorphic bands distinguished the salt-tolerant-selected lines from control. For example, bands with molecular weights of 690 and those of 950, 750 and 200 bp were detected only in salinity-tolerant selected shoot lines when UBC811 and ISSR3 primers were used, respectively (Figure S6).

## 3. Discussion

Exogenous and endogenous growth regulators interact to exert metabolic and morphological effects. In paulownia, poor shoot multiplication was detected on basal MS medium, MS supplemented with low (1 mg/L) or high concentrations of BAP (more than 2 mg/L). While the considerable increase in shoot number was detected when explants were cultured on MS medium containing 2 mg/L BAP, the interaction between 2 mg/L BAP and 0.1 NAA improved the results, but it was affected by the water potential of the medium. Media with lower water potential (MS and WPM) were better than others (SH and B5). Generally, the number of formed shoots and their growth are mainly dependent on the concentration of the used growth regulators and medium type [28,29].

When different media with 3% sucrose were used, different water potentials (MS (−435 MPa), SH (−376 MPa), B5 (−366 MPa) and WPM (−427 MPa)) were created [15,30]. Regardless of the type of medium used, the interaction between 2 mg/L BAP and 0.1 mg/L NAA resulted in the highest number of formed shoots. The magnification of the effect of interaction between 2 mg/L BAP and 0.1 mg/L NAA was required for the establishment of Ψ that was created by MS or WPM media. MS was the best. In several plant species, shoot multiplication and growth needed MS medium [22,31].

To confirm the interaction between water potential of the medium and growth regulators during differentiation and growth, liquid, semisolid and solid MS agar media were used. Water potentials of MS media solidified with agar in semisolid and solid forms had more negative Ψ than that of a liquid medium [32]. Consequently, the interaction between 2 mg/L BAP and 0.1 mg/L NAA, and Ψ generated by the nutrient MS medium in semisolid or solid form was necessary for better shoot multiplication. Additionally, the fundamental changes in medium Ψ resulting from the use of half- or double-strength MS medium exerted a significant negative effect on the ability of the used growth regulators for differentiation and growth of the formed shoots [28].

Changing the cytokinin type resulted in changes in the recorded data. When 2 mg/L of BAP was substituted with 2 mg/L of Kin and or 2-ip, shoot multiplication and growth decreased. In many woody species, BAP is more effective than Kin for induction of shoot formation [33]. Additionally, in other works, the application of suitable concentration of BAP resulted in the highest shoot multiplication rates when compared to that of Kin and Zeatin [34,35], but in the *Melia azedarach* tree, Kin was more effective than BAP [36].

The interaction between 0.1 mg/L GA and 0.1 mg/L NAA with 2 mg/L BAP modulated the obtained data. For example, in a medium containing 2 mg/L BAP, while the incorporation of 0.1 mg/L NAA improved multiplication and growth, but 0.1 mg/L GA retard them. This retardation disappeared when the three growth regulators were included. Leite et al. [37] reported that when gibberellin and cytokinin were applied separately, each of them promoted shoot growth but only when they were applied together, and cytokinin inhibited the effects of gibberellin, leading to a reduction in the plant size, number of nodes, stem diameter, leaf area and dry matter. In this work, the presence of GA alone or in combination with BAP or NAA increased the length of the internode but decreased leaf area. De Mason et al. [38] reported that both auxin and GA play similar and significant roles in leaf development in peas, indicating the interaction between the action of both of them [39]. In paulownia, the detected changes due to the exogenous application of GA (decrease in leaf area, stem diameter and shoot fresh weight) may be due to an indirect increase in auxin concentration, leading to the modulation of the auxin/cytokinin ratio [40] or an increase in auxin synthesis in leaves [41]. The application of GA was able to modulate the expression of several auxin signaling genes [42]. In addition, the detected reduction in plant leaves of paulownia may be due to the increase in auxin and gibberellin contents that negatively affect the cytokinin/auxin ratio [9]. It was reported that the growth capacity of the leaves is proportional to their content of GAs [8]. In paulownia, it seems that the sensitivity of leaves to GAs was higher than that of internodes. A negative interaction between gibberellin and cytokinin on elongation of the bean stem was detected [43]. In paulownia, the presence of BAP with NAA and GA reduced the negative interaction of BAP on GA due to the creation of new interactions between three GRs. It means that hormonal interaction determines the final outcome of the individual hormone actions as was reported by [44]. 

The promotion of growth by gibberellins takes place through the activation of hydrolytic enzymes that increases the length of the cells compared to their diameter, making tissues and organs (leaves, stems or fruits) longer and thinner [45,46]. In paulownia, the effect of GA on growth needed the presence of BAP and NAA. Consequently, MS medium containing only GA showed low values of shoot multiplication and shoot growth. This was associated with low staining density of the detected bands and disappearance of other bands (EST-1 and EST-6), indicating a decrease in esterase activity [47]. This situation was reversed when shoots multiplied on MS medium containing 2 mg/L BAP and 0.1 mg/L GA were characterized by an increase in the staining intensity of the detected bands and appearance of one new band (EST-1) regardless of the presence or absence of 0.1 mg/L NAA. It increased the role of esterases in the cell wall elongation of paulownia shoots [11].

The reduction in leaf area and stem diameter and increase in internode length facilitated the formation and conversion of paulownia synseeds. The encapsulation of small plant materials resulted in the formation of consistently shaped beads that are easy to handle [19]. In addition, the reduction in leaf blade size facilitated the observation of cultures and subcultures, as was noticed by [48].

In paulownia, efficient root formation was obtained when 1 mg/L IBA was used, especially in half-strength MS medium. The addition of 1 mg/L NAA in a half-strength MS medium was better than 1 mg/L IBA. The best results were obtained by using 0.5 mg/L of NAA and IBA each. The combination of two auxins was more effective for the induction of root formation than only one type [49,50]. Rooted plantlets were successfully acclimatized to ex vitro conditions [51]. 

Shoot cuttings transferred from multiplication medium containing 2 mg/L BAP, 0.1 mg/L NAA and 0.1 mg/L GA to root induction medium (MS medium with 0.5 mg/L IBA and 0.5 mg/L NAA) still contain GA, leading to decreased root formation. Kumar et al. [50] found that the incorporation of gibberellins in rooting medium reduced or prevented the formation of adventitious roots. The negative effect of GA on root formation, as well as a decrease in stem diameter and leaf area, led to the decrease in the percentage of plants that passed the acclimatization stage. Leaves and roots sizes are crucial for plant survival and growth, but they are negatively affected by GA supply [52]. The choice of optimal medium composition for the formation of an extensive root system was an essential prerequisite to improving the ex vitro acclimatization of micropropagated plants [51].

Five in vitro selected salt-tolerant lines were obtained from nodal explants subjected to the applied selection procedure under the influence of long-term culture on semisolid MS medium and the interaction between 2 mg/L BAP, 0,1 mg/L NAA and 0.1 mg/L GA. Only the selected NaCl-tolerant shoot cuttings were able to survive, whereas microshoots that were not subjected to NaCl showed 100% mortality, as was previously reported [53]. The growth of the selected lines was affected by saline conditions to a lesser extent than the others, where they conserved their ability to multiply and grow [54]. 

Under long-term culture, while the number of salt-tolerant selected lines (five lines) is 50% less than the number of lines tested under non-selection conditions (ten lines), the genetic variance increased from 40.3% to 46% in the case of RAPD and from 49.02% to 51.4% in the case of ISSR. In paulownia, no polymorphic DNA fragments were detected among the micropropagated plants in correlation to the mother plant where all the detected bands were monomorphic patterns [55]. This gave clear evidence that long-term culture increased genetic variation in paulownia and other plant species [23,56]. The genetic variation resulting from the interaction between 2 mg/L BAP, 0.1 mg/L NAA and 0.1 mg/L GA and Ψ of semisolid MS medium for long-term culture and NaCl was used to improve the plants’ ability to resist harsh environmental conditions, such as salinity. Both RAPD and ISSR were used for the detection of genetic variation under these and other conditions [22,23,47,57]. The pattern of the five selected shoot lines was distinguished from that of control by the appearance of new bands of 610 and 300 bp using RAPD primers OPA-07 and OPG-02, respectively. Additionally, distinguishing the selected salt-tolerant plants from the control plants was possible using ISSR. It is worth noting that the values of genetic variance that resulted from the use of ISSR were higher than the use of RAPD. The detected genetic variation may be due to the presence of hot spots in the nuclear genome that transposable elements can jump around, leading to genetic changes in in vitro obtained plants [58]. The dominant genetic markers, such as ISSR and RAPD, have a great potential to reveal the genetic variation between plant species [59].

## 4. Materials and Methods

### 4.1. Plant Material and Shoot Culture Establishment

Shoots of paulownia hybrid (*P. tomentosa* × *P. elongata*) were brought from Woody Tree Research Department at the Agricultural Research Center, Giza, Egypt. For disinfection, stem segments (about 3–5 cm length) with about 2 nodes were immersed in Clorox (5% *w/v*) for 5 min and HgCl_2_ (0.2% *w/v*) for 2 min and then washed three times (for 5, 10, and 15 min) with sterilized distilled water. Then, about 1 cm long explants with one node were obtained and cultured on MS medium [60] containing 2 mg/L BAP. Cultures were incubated for one month in the tissue culture room at 26 ± 2 °C, 16/8 h photoperiod with an irradiance of 100 µmol m^−2^ s^−1^, and 70–80% humidity. In vitro obtained shoots were subcultured to obtain enough plant material to examine the impact of several factors on paulownia micropropagation. Generally, in all multiplication experiments, four replicates/treatment, each with ten nodal explants (about 0.5 cm length) were cultured for one month to determine the frequency of shoot formation (%), shoot number (No.) per explant, shoot length (cm), node number per shoot, leaves per shoot, fresh mass (F.w.)/shoot cluster (g) and fresh mass/one shoot (g).

### 4.2. Determination of the Best BAP Concentration for Paulownia Multiplication and Growth

To determine the best BAP concentration for paulownia multiplication and growth, nodal explants were cultured on MS medium with different BAP concentrations (0, 1, 2, 3 or 5 mg/L).

### 4.3. Interaction between Selected Concentration of BAP and NAA and Different Growth Media with Different Water Potentials

In vitro grown shoots were cut into nodal explants and cultured on different basal media (MS, B5 (Gamborg B5 medium [61], SH [62] or woody plant medium (WPM)) [63]) with different water potential supplemented with 2 mg/L BAP and different concentrations (0, 0.1 or 0.5 mg/L) of NAA.

### 4.4. Interaction between Selected Concentration of BAP and NAA in MS Medium Solidified with Different Agar Concentrations Creating Different Water Potentials

To study the interaction between selected concentrations of BAP and NAA and different strengths of MS medium with different water potentials, in vitro nodal segments were cultured on MS medium containing 2 mg/L BAP + 0.1 mg/L NAA to be liquid (0 g/L agar), semisolid (4 g/L agar) or solid (8 g/L agar). 

### 4.5. Interaction between Selected Concentration of BAP and NAA and Different Strengths of MS Medium with Different Water Potentials

In vitro nodal segments were cultured on half-, full- or double-strength MS medium containing 2 mg/L BAP + 0.1 mg/L NAA (each was solidified with 4 g/L agar). 

### 4.6. Interaction between NAA and Different Types of Cytokinins on Paulownia Multiplication and Growth

Microshoots were cut into nodal segments and cultured on semisolid MS medium containing 0.1 mg/L NAA and 2 mg/L of different cytokinins (BAP, Kin or 2-ip). 

### 4.7. Interaction between Selected Concentration of BAP and NAA and Different Concentrations of GA

In vitro obtained nodal segments were cultured on semisolid MS medium supplemented with 2 mg/L BAP and 0.1 mg/L NAA, and different concentrations (0, 0.1, 0.25, 1 or 2 mg/L) of GA. The best concentration of GA (0.1 mg/L) was used alone or in combination with 0.1 mg/L NAA and different concentrations of BAP (2, 3, or 4 mg/L) to detect the effect of growth regulator combination on shoot elongation and leaf area (Table 6). In vitro shoots grown on MS medium with 2 mg/L BAP and 0.1 mg/L NAA with or without 0.1 mg/L GA, MS medium with 0.1 mg/L GA or MS medium with 2 mg/L BAP and 0.1 mg/L GA were subjected to esterase analysis by gel electrophoresis techniques.

### 4.8. Synseed Conversion as Influenced by GRs Interaction on the Source of Plant Materials

In vitro nodal segments were excised from microshoots grown on MS medium fulfilled with 2 mg/L BAP and 0.1 mg/L NAA with or without 0.1 mg/L GA (plant material source) and encapsulated in single layer beads, as described by Hassanein et al. [20]. Liquid MS with 2 mg/L BAP and 0.1 mg/L NAA was used for synthetic endosperm synthesis. Encapsulation was carried out using an MS medium containing 3% *w/v* of sodium alginate (gelling matrix) and 75 mM CaCl2 solution (complexing agent). Synseeds were conserved in a thin layer of a liquid MS medium with 2 mg/L BAP and 0.1 mg/L NAA at 4 °C in the refrigerator for one month. For conversion, synseeds were placed on semisolid MS with 2 mg/L BAP and 0.1 mg/L NAA and incubated under tissue culture room conditions for one more month.

### 4.9. In Vitro Root Formation under the Effect of Different IBA Concentrations 

In all experiments of root formation, four replicates/treatment, each with ten microshoots (about 1.5–2 cm length), were achieved and incubated under tissue culture room conditions, and the following parameters were registered: frequency of root formation, the number of roots/shoot and length of the root system (cm). To study the root formation as influenced by different concentrations of IBA, microshoots were cultured for three weeks on semisolid full-strength MS medium augmented with various concentrations (0, 0.5, 1 or 2 mg/L) of IBA.

### 4.10. In Vitro Rooting as Influenced by Different Auxin Types

Microshoots were cultured for three weeks on semisolid half-strength MS media appended with: 1 mg/L of different auxins (NAA or IAA) or 0.5 mg/L IBA + 0.5 mg/L NAA.

### 4.11. In Vitro Rooting under the Influence of GRs Interaction

Microshoots grown on semisolid MS medium supplemented with 2 mg/L BAP and 0.1 mg/L NAA with or without 0.1 mg/L GA for one month were cultured for three weeks on a semisolid half-strength MS medium containing 0.5 mg/L IBA and 0.5 mg/L NAA.

### 4.12. Transfer of Plantlets to Soil and Acclimatization

Plantlets with a well-formed root system were transferred individually to plastic cups filled with a mixture of sand and peat moss (1:2 *v/v*) and covered by transparent polyethylene bags to guarantee high humidity. After one week, the bags were gradually poured to reduce the humidity and were completely removed after three weeks. They were acclimatized gradually to the open field and then were transferred outdoors under full sun.

### 4.13. Determination of Sublethal Concentration of NaCl

Nodal explants (about 0.5 cm length) obtained from in vitro grown shoots were cultured on MS with 2 mg/L BAP, 0.1 mg/L NAA and different concentrations (0, 50, 100, 150, 175 or 200 mM) of NaCl for one month to determine the sublethal concentration of NaCl (175 mM). The lethal concentration was a certain concentration of NaCl in a semisolid MS medium, which resulted in a Ψ that completely prevents the multiplication and growth of subcultured shoot cuttings and may lead to death on a large scale. The concentration below the lethal concentration was considered to be the sublethal concentration.

### 4.14. Induction of Genetic Variation through Long Term Culture

For the induction of somatic variation, shoots from ten different jars were subcultured on a new semisolid MS medium with 2 mg/L BAP and 0.1 mg/L NAA fourteen times, one month each. Then, shoots were subjected for DNA extraction and analyzed via RAPD-PCR and ISSR-PCR techniques 

### 4.15. Selection of Salt-Tolerant Shoot Lines

To select a salt-tolerant variant, 85 shoot lines that were exposed to a long-term culture for eight subcultures on a semisolid MS medium containing 2 mg/L BAP, 0.1 mg/L NAA and 0.1 mg/L GA were transferred (9th subculture) to the same medium in addition to 50 mM NaCl for one month. Then, shoots were subcultured on a new medium with 100 mM NaCl (10th subculture) for one month. Finally, shoots were subcultured on a new medium with 175 mM NaCl (11th subculture) for one month. Ultimately, ten shoot lines were selected and subcultured on a new medium without NaCl for two successive passages (12th and 13th subcultures), one month each. Thereafter, they were returned again to a new medium with 175 mM NaCl for one month to select the more salt-tolerant shoots to breed them. Five lines were selected and subjected to DNA extraction for RAPD-PCR and ISSR-PCR analysis in comparison to one shoot not absolutely subjected to NaCl (control).

### 4.16. Isoenzymes Analysis

Two grams of paulownia microshoots were homogenized at 4 °C in 1 mL of extraction buffer containing 0.2 μM Tris-HCl, pH 7.0 and 0.004 M cysteine. The obtained homogenate was centrifuged at 13 rpm and 4 °C for 15 min. and collected on 7.5% (*w/v*) polyacrylamide slab gels for electrophoresis. During electrophoresis, a run buffer containing 0.025 M Tris-base and 0.192 M glycine at an 8.9 pH was used. The run was completed in 6 h at 8 mA and 10 °C. Esterase (EST) was stained, as described by Brewer [64].

### 4.17. DNA Extraction

Genomic DNAs were extracted from paulownia in vitro grown shoots by Cetyltrimethylammonium bromide method (modified by Porebski et al. [65]). 

### 4.18. RAPD-PCR Analysis

Ten random 10-mer primers (OPA-02, OPA-03, OPA-06, OPA-07, OPA-08, OPA-15, OPH-16, OPBC-13, OPG-02 and OPR11) were used. Genomic DNAs were extracted from paulownia in vitro grown shoots that were subjected to long-term culture with or without NaCl. Polymerase chain reactions (PCR) were fulfilled in 25 µL end volume, each comprised 6.5 µL deionized H_2_O, 12.5 µL master mix, 3 µL primer and 3 µL template DNA. Amplification processes were conducted in a Perkin Elmer/GeneAmp PCR System 9700 (PE Applied Biosystems). System was programmed to accomplish 40 cycles after an initial cycle (denaturation at 94 °C for 5 min). Each cycle contained denaturation (45 s at 94 °C), annealing (50 s at 36 °C) and elongation (1 min at 72 °C). Then, primer segments’ extensions were extended to 7 min at 72 °C.

### 4.19. ISSR-PCR Analysis

Template DNAs of ten paulownia microshoots subjected to long-term culture or five salt-tolerant lines were amplified by ten ISSR primers (ISSR-1, ISSR-2, ISSR-3, ISSR-5, ISSR-6, ISSR-14, UBC808, UBC811, UBC817 and 844 A (or 844 B)). Amplification reactions were fulfilled in 25 μL end volumes comprised the same components of RAPD reactions except for the primers that were replaced by ISSR primers. Amplification conditions were the same as RAPD conditions except that the annealing temperature was at 48 °C for 1 min.

### 4.20. DNA Fragments’ Visualization

All PCR products were visualized via horizontal gel electrophoresis on 1.5% (*w/v*) agarose gel containing 0.5 μg/mL ethidium bromide in 1× TBE buffer (750 mM Tris-HCl, 900 mM boric acid and 2 mM Na_2_-EDTA). Electrophoresis was performed under a constant voltage of 80 V for 2 h. The obtained DNA patterns were analyzed by the MVSP computer software program of Nei and Li [66].

### 4.21. Statistical Analysis

All data were analyzed and expressed as average ± SD (standard deviation) according to Snedecor and Cochran [67]. Additionally, data were analyzed by one-way analysis of variance (ANOVA) using SPSS 16. The level of significance was measured by running a Tukey test at *p* < 0.05 level of significance.

## 5. Conclusions

The production of paulownia tree tolerant to or free from pathogens with the possibility of its in vitro multiplication was obtained [24,25,26,27]. For true to type-mass multiplication or improvement, a deep understanding of the interaction between growth regulators and the Ψ of the growth environment was determined. In paulownia, the interaction between 2 mg/L BAP and 0.1 mg/L NAA and Ψ generated by the MS medium in semisolid form was necessary for better shoot multiplication. When these two GRs interacted with 0.1 mg/L GA, leaf area, stem diameter and shoot fresh weight of the formed shoots decreased, which facilitated the formation of synseeds and their conversion. Efficient root formation was obtained when 0.5 mg/L of each of NAA and IBA was used. Shoot cuttings transferred from shoot multiplication medium (semisolid MS medium with 2 mg/L BAP, 0.1 mg/L NAA and 0.1 mg/L GA) to root induction medium still contain GA, leading to decreased root formation and acclimatization. While no polymorphism was detected among the micropropagated paulownia [55], long-term culture under created interaction (2 mg/L BAP, 0.1 mg/L NAA, 0.1 mg/L GA, Ψ of semisolid MS medium, long term culture condition and NaCl) increased genetic variation as other plant species [23,56] and used to improve the plant ability to tolerate salt stress. Consequently, the interaction between the used growth regulators and Ψ of the in vitro multiplication environment of the paulownia tree was clear. Therefore, studies of the interaction between these items on in vitro multiplication and improvement should be conducted on other plant species.

## Figures and Tables

**Table 1:** Effect of different BAP concentrations in MS medium on in vitro shoot multiplication and growth of paulownia for one month.

BAP Conc. (mg/L)	No. of Shoots/Explant	Length of Shoot (cm)	No. of Nodes/Shoot	No. of Leaves/Shoot	F.W./Shoot Cluster (g)
0	1.7 ± 0.6 ^b^	1.2 ± 0.1 ^c^	4 ± 1 ^a^	8 ± 2 ^a^	0.059 ± 0.01 ^c^
0.5	2 ± 0 ^b^	1.6 ± 0.4 ^b^	4 ± 0 ^a^	8.7 ± 1.2 ^a^	0.076 ± 0.01 ^b^
1	2 ± 0 ^b^	1.8 ± 0.1 ^a^	4 ± 0 ^a^	9.3 ± 1.2 ^a^	0.083 ± 0.01 ^b^
2	3.7 ± 0.6 ^a^	2.2 ± 0.06 ^a^	4.3 ± 0.6 ^a^	10.7 ± 1.2 ^a^	0.125 ± 0.01 ^a^
3	2.3 ± 0.6 ^b^	1.3 ± 0.2 ^c^	4.3 ± 0.6 ^a^	9.3 ± 1.2 ^a^	0.090 ± 0.01 ^b^
5	2 ± 0 ^b^	1.6 ± 0.06 ^b^	4.3 ± 0.6 ^a^	8.6 ± 1.2 ^a^	0.079 ± 0.001 ^b^

Values are mean ± SD. Values not followed by the same letter are significantly different at *p* ˂ 0.05; Tukey’s test was within the same parameter.

**Table 2:** Effect of different NAA concentrations in combination with 2 mg/L BAP in different basal media types on in vitro shoot multiplication and growth of paulownia for one month.

Type of Basal Medium	NAA Conc. (mg/L)	No. of Shoots/Explant	Length of Shoot (cm)	No. of Nodes/Shoot	No. of Leaves/Shoot	F.W./Shoot Cluster (g)	Callus Formation
MS	0	3.7 ± 0.6 ^b^	2.2 ± 0.06 ^c^	4.3 ± 0.6 ^a^	10.7 ± 1.2 ^a^	0.125 ± 0.01 ^c^	++
	0.1	4.3 ± 0.6 ^a^	2.9 ± 0.2 ^a^	4.3 ± 0.6 ^a^	10.7 ± 1.2 ^a^	0.241 ± 0.04 ^a^	++
	0.5	2.7 ± 0.6 ^d^	2.4 ± 0.1 ^b^	4 ± 0 ^b^	9.7 ± 0.6 ^b^	0.142 ± 0.02 ^b^	++
B5	0	2 ± 0 ^e^	1.8 ± 0.2 ^e^	3.3 ± 0.6 ^c^	7.7 ± 0.6 ^f^	0.066 ± 0.004 ^e^	+
	0.1	3.3 ± 0.6 ^c^	2.2 ± 0.2 ^c^	4 ± 0 ^b^	8.7 ± 1.2 ^d^	0.123 ± 0.01 ^c^	+
	0.5	2.3 ± 0.6 ^d^	2.1 ± 0.2 ^c^	3 ± 0 ^d^	8 ± 0 ^e^	0.120 ± 0.01 ^c^	+
SH	0	1.7± 0.6 ^e^	1.8 ± 0.1 ^e^	2.7 ± 0.6 ^e^	6.7 ± 1.2 ^g^	0.099 ± 0.01 ^d^	−
	0.1	2 ± 0 ^e^	1.8 ± 0.4 ^e^	3.3 ± 0.6 ^c^	7.3 ± 1.2 ^f^	0.100 ± 0.01 ^d^	−
	0.5	2 ± 0 ^e^	1.7 ± 0.06 ^f^	2.3 ± 0.6 ^e^	5.3 ± 0.6 ^g^	0.063 ± 0.01 ^e^	−
Wm	0	3.3 ± 0.6 ^c^	2 ± 0.1 ^d^	4 ± 0 ^b^	9.3 ± 1.2 ^c^	0.189 ± 0.03 ^b^	+++
	0.1	4 ± 0 ^a^	2 ± 0.1 ^d^	4 ± 0 ^b^	9.3 ± 1.2 ^c^	0.229 ± 0.07 ^a^	+++
	0.5	2.7 ± 0.6 ^d^	2 ± 0.1 ^d^	3.3 ± 0.6 ^c^	8 ± 0 ^e^	0.160 ± 0.03 ^b^	+++

+ = small callus, ++ = moderate callus, +++ = large callus, − = no callus. Values are mean ± SD. Values not followed by the same letter are significantly different at *p* ˂ 0.05; Tukey’s test was within the same parameter.

**Table 3:** Effect of different textures of MS medium containing 2 mg/L BAP and 0.1 mg/L NAA on shoot multiplication and growth of paulownia for one month.

Agar	Texture of Medium	No. of Shoots/Explant	Length of Shoot (cm)	No. of Nodes/Shoot	No. of Leaves/Shoot	F.W./Shoot Cluster (g)
0	Liquid	2 ± 0 ^b^	0.2 ± 0.1 ^b^	1 ± 0 ^c^	2 ± 0 ^c^	0.015 ± 0.001 ^c^
4	Semisolid (Control)	4.7 ± 0.6 ^a^	3.2 ± 0.2 ^a^	5.7 ± 0.6 ^a^	13.3 ± 1.2 ^a^	0.626 ± 0.06 ^a^
8	Solid	4.3 ± 0.6 ^a^	2.9 ± 0.2 ^a^	4.3 ± 0.6 ^b^	10.7 ± 1.2 ^b^	0.241 ± 0.04 ^b^

Values are mean ± SD. Values not followed by the same letter are significantly different at *p* ˂ 0.05; Tukey’s test was within the same parameter.

**Table 4:** Effect of different strengths of semisolid MS medium containing 2 mg/L BAP and 0.1 mg/L NAA on shoot multiplication and growth of paulownia for one month. Values are means ± SD.

Strength of MS Basal Medium	No. of Shoots/Explant	Length of Shoot (cm)	No. of Nodes/Shoot	No. of Leaves/Shoot	F.W./Shoot Cluster (g)
Half strength	2.7 ± 0.6 ^b^	1.6 ± 0.06 ^c^	2.7 ± 0.6 ^c^	7.3 ± 1.2 ^b^	0.14 ± 0.02 ^b^
Full strength (control)	4.7 ± 0.6 ^a^	3.2 ± 0.2 ^a^	5.7 ± 0.6 ^a^	13.3 ± 1.2 ^a^	0.63 ± 0.06 ^a^
Double strength	1.7 ± 0.6 ^b^	2.6 ± 0.3 ^b^	4.7 ± 0.6 ^b^	10 ± 2 ^a^	0.17 ± 0.0.01 ^b^

Values are mean ± SD. Values not followed by the same letter are significantly different at *p* ˂ 0.05; Tukey’s test was within the same parameter.

**Table 5:** Effect of 2 mg/L of different cytokinins in combination with 0.1 mg/L NAA in semisolid MS medium on paulownia in vitro shoot multiplication and growth for one month.

Type of Cytokinin	No. of Shoots/Explant	Length of Shoot (cm)	No. of Nodes/Shoot	No. of Leaves/Shoot	F.W./Shoot Cluster (g)
BAP	4.7 ± 0.6 ^a^	3.2 ± 0.2 ^a^	5.7 ± 0.6 ^a^	13.3 ± 1.2 ^a^	0.626 ± 0.06 ^a^
Kin	1.7 ± 0.6 ^b^	2.9 ± 0.06 ^a^	4.3 ± 0.6 ^b^	8.6 ± 1.2 ^b^	0.091 ± 0.01 ^b^
2-ip	2 ± 0 ^b^	2.03 ± 0.1 ^b^	3 ± 0 ^c^	7 ± 1 ^c^	0.089 ± 0.01 ^b^

Values are mean ± SD. Values not followed by the same letter are significantly different at *p* ˂ 0.05; Tukey’s test was within the same parameter.

**Table 6:** Effect of different concentrations of GA alone or in combination with 0.1 mg/L NAA and different concentrations of BAP in semisolid MS medium on in vitro shoot multiplication and growth of paulownia.

Type of Hormone			No. of Shoots/ Explant	Length of Shoot (cm)	No. of Nodes/ Shoot	No. of Leaves/ Shoot	Length of Internode (cm)	F.W./ Shoot Cluster (g)	F.W./One Shoot (g)	Leaf Area (cm^2^)
BAP (mg/L)	NAA (mg/L)	GA (mg/L)								
2	0.1	-	4.7 ± 0.6 ^a^	3.2 ± 0.2 ^b^	5.7 ± 0.6 ^a^	13.3 ± 1.2 ^a^	0.5 ± 0.06 ^c^	0.626 ± 0.06 ^a^	0.05 ± 0.01 ^a^	0.88 ± 0.12 ^a^
2	-	0.1	3 ± 1 ^c^	4.5 ± 0.1 ^a^	4.3 ± 0.6 ^b^	9.3 ± 1.2 ^a^	1.6 ± 0.1 ^a^	0.142 ± 0.01 ^b^	0.039 ± 0.002 ^a^	0.32 ± 0.04 ^b^
-	-	0.1	1.7 ± 0.6 ^e^	1.3 ± 0.1 ^d^	2.3 ± 0.6 ^d^	5.3 ± 0.6 ^b^	0.5 ± 0.06 ^c^	0.041 ± 0.003 ^c^	0.011± 0.001 ^d^	0.11 ± 0.03 ^d^
2	0.1	0.1	4.3 ± 0.6 ^b^	5.03 ± 0.5 ^a^	5.7 ± 0.6 ^a^	10.7 ± 1.2 ^a^	1.8 ± 0.1 ^a^	0.188 ± 0.04 ^b^	0.035 ± 0.003 ^b^	0.34 ± 0.03 ^b^
3	0.1	0.1	4.3 ± 0.6 ^b^	3.3 ± 0.2 ^b^	4.7 ± 0.6 ^b^	11.3 ± 1.2 ^a^	0.8 ± 0.02 ^b^	0.167 ± 0.02 ^b^	0.016 ± 0.003 ^d^	0.11 ± 0.03 ^d^
4	0.1	0.1	4.3 ± 0.6 ^b^	2.3 ± 0.3 ^c^	4.3 ± 0.6 ^b^	9.3 ± 1.2 ^a^	0.7 ± 0.06 ^b^	0.151 ± 0.01 ^b^	0.014 ± 0.002 ^d^	0.10 ± 0.03 ^a^
2	0.1	0.25	3.3 ± 0.6 ^c^	4.6 ± 0.1 ^a^	4.7 ± 0.6 ^b^	9.3 ± 1.2 ^a^	1.1 ± 0.1 ^b^	0.124 ± 0.05 ^b^	0.024 ± 0.002 ^c^	0.31 ± 0.02 ^b^
2	0.1	0.5	2.7 ± 0.6 ^d^	3.2 ± 0.2 ^b^	3 ± 1 ^c^	5.3 ± 1.2 ^b^	0.9 ± 0.2 ^b^	0.111 ± 0.02 ^b^	0.014 ± 0.001 ^d^	0.21 ± 0.04 ^c^
2	0.1	1	2.3 ± 0.6 ^e^	1.7 ± 0.1 ^d^	2.3 ± 0.6 ^d^	4.7 ± 1.2 ^b^	0.6 ± 0.1 ^c^	0.158 ± 0.04 ^b^	0.007 ± 0.002 ^e^	0.11 ± 0.03 ^d^
2	0.1	2	2.3 ± 0.6 ^e^	1.5 ± 0.06 ^d^	2.3 ± 0.6 ^d^	4 ± 0 ^b^	0.4 ± 0.1 ^c^	0.126 ± 0.03 ^b^	0.004 ± 0.001 ^e^	0.10 ± 0.02 ^d^

Values are mean ± SD. Values not followed by the same letter are significantly different at *p* ˂ 0.05; Tukey’s test was within the same parameter.

**Table 7:** Effect of presence or absence of GA in plant materials medium on conversion of conserved (for one month at 4 °C) paulownia synseeds on semisolid MS medium containing 2 mg/L BAP and 0.1 mg/L NAA for one month.

Medium from Which Encapsulated Plant Materials Were Obtained	% of Shoot Formation	No. of Shoots/Explant	Length of Shoot (cm)	No. of Nodes/Shoot	No. of Leaves/Shoot	F.W./Shoot Cluster (g)
MS + 2 mg/L BAP + 0.1 mg/L NAA	70 ^b^	2.3 ± 0.6 ^b^	2.4 ± 0.1 ^a^	4.3 ± 0.6 ^a^	10.7 ± 1.2 ^a^	0.38 ± 0.05 ^a^
MS + 2 mg/L BAP, 0.1 mg/L NAA + 0.1 mg/L GA	93.3 ^a^	3.7 ± 0.6 ^a^	2.8 ± 0.06 ^a^	5.7 ± 0.6 ^a^	11.3 ± 1.2 ^a^	0.25 ± 0.01 ^b^

Values are mean ± SD. Values not followed by the same letter are significantly different at *p* ˂ 0.05, Tukey’s test was within the same parameter.

**Table 8:** Root formation and growth on paulownia microshoots cultured on semisolid full-strength MS medium containing different IBA concentrations or semisolid half-strength MS medium containing 1 mg/L of different auxins for three weeks.

Strength of MS Medium	Auxin Type and Concentration (mg/L)			Frequency of Root Formation (%)	No. of Roots/Shoot	Length of Root System (cm)
	IBA	NAA	IAA			
**Full**	-	-	-	60 ^d^	2.7 ± 0.6 ^d^	1.8 ± 0.1 ^e^
	0.5	-	-	83.3 ^c^	3.3 ± 0.6 ^d^	2.6 ± 0.3 ^d^
	1	-	-	93.3 ^b^	5 ± 1 ^c^	4.4 ± 0.3 ^b^
	2	-	-	90 ^b^	3.7 ± 1.2 ^c^	3.6 ± 0.7 ^c^
**Half**	1	-	-	100 ^a^	9 ± 1 ^b^	4.2 ± 0.3 ^b^
	-	1	-	100 ^a^	11 ± 1 ^b^	6.4 ± 1.4 ^a^
	-	-	1	20 ^e^	1.3 ± 0.6 ^e^	1 ± 0.3 ^e^
	0.5	0.5	-	100 ^a^	14.3 ± 1.2 ^a^	5.5 ± 0.5 ^a^

Values are mean ± SD. Values not followed by the same letter are significantly different at *p* ˂ 0.05; Tukey’s test was within the same parameter.

**Table 9:** Root formation and growth as influenced by GRs interaction and culture for three weeks on semisolid half-strength MS medium containing 0.5 mg/L IBA and 0.5 mg/L NAA.

Medium from Which Microshoots Were Obtained	Frequency of Root Formation (%)	No. of Roots/Shoot	Length of Root System (cm)
2 mg/L BAP and 0.1 mg/L NAA (Control)	100 ^a^	14.3 ± 1.5 ^a^	5.5 ± 0.5 ^a^
2 mg/L BAP, 0.1 mg/L NAA and 0.1 mg/L GA	93.3 ^a^	9.3 ± 1.5 ^b^	1.7 ± 0.2 ^b^

Values are mean ± SD. Values not followed by the same letter are significantly different at *p* ˂ 0.05; Tukey’s test was within the same parameter.

**Table 10:** RAPD and ISSR analysis to determine somaclonal variation among different paulownia lines as influenced by long-term culture on MS medium containing 2 mg/L BAP and 0.1 mg/L NAA for one month.

Primer Type	Primer Code	No. of Total Scorable Fragments	Size of Amplified Fragments (bp)	No. of Polymorphic Bands	No of Monomorphic Bands	No. of Unique Bands	Polymorphism (%)
RAPD	OPA-02	11	100–950	1	9	1	18.2
	OPA-03	4	270–700	1	3	0	25
	OPA-06	5	250–1200	3	2	0	60
	OPA-07	12	125–950	2	9	1	25
	OPA-08	3	180–450	2	1	0	66.7
	OPA-15	8	230–1300	2	6	0	25
	OPH-16	5	240–950	3	1	1	80
	OPBC-13	6	350–1050	1	4	1	33.3
	OPG-02	11	130–1100	5	6	0	45.5
	OPR-11	7	350–1150	4	2	1	71.4
	**Total**	**72**		**24**	**43**	**5**	**40.3**
ISSR	ISSR-1	7	200–500	5	0	2	100
	ISSR-2	6	250–1000	3	2	1	66.7
	ISSR-3	7	250–950	4	3	0	62.5
	ISSR-5	2	480–1000	0	2	0	0
	ISSR-6	8	225–850	5	3	0	62.5
	ISSR-14	2	400–500	0	2	0	0
	UBC808	5	250–550	1	4	0	20
	UBC811	9	200–650	1	7	1	22.2
	UBC817	3	390–470	2	1	0	33.3
	844 A	2	210–340	0	2	0	0
	**Total**	**51**		**21**	**26**	**4**	**49.02**

**Table 11:** RAPD and ISSR analysis to detect somaclonal variation among different NaCl-tolerant selected lines obtained from plants subjected to long-term culture and NaCl on semisolid MS medium containing 2 mg/L BAP and 0.1 mg/L NAA. Control shoot cutting was also included.

Primer Type	Primer Code	No. of Total Scorable Fragments	Size of Amplified Fragments (bp)	No. of Polymorphic Bands	No of Monomorphic Bands	No. of Unique Bands	Polymorphism (%)
RAPD	OPA-02	10	100–900	6	1	3	90
	OPA-03	8	120–900	0	8	0	0
	OPA-06	2	390–750	0	2	0	0
	OPA-07	3	380–700	1	2	0	33.3
	OPA-08	2	175–450	1	1	0	50
	OPA-15	4	350–750	0	2	2	50
	OPH-16	2	225–650	1	1	0	50
	OPBC-13	8	225–1500	4	3	1	62.5
	OPG-02	7	148–500	2	4	1	42.9
	OPR-11	4	500–1100	0	3	1	25
	**Total**	**50**		**15**	**27**	**8**	**46**
ISSR	ISSR-1	9	225–650	4	4	1	55.6
	ISSR-2	4	350–950	2	1	1	75
	ISSR-3	10	200–950	6	4	0	60
	ISSR-5	4	450–1200	3	1	0	75
	ISSR-6	9	210–850	4	4	1	50
	ISSR-14	5	230–520	2	2	1	60
	UBC808	6	250–650	2	4	0	33.3
	UBC811	8	180–680	3	5	0	37.5
	UBC817	8	275–750	3	5	0	37.5
	844 B	9	200–950	3	5	1	44.4
	**Total**	**72**		**32**	**35**	**5**	**51.4**

## Data Availability

The datasets generated and/or analyzed during the current study are available from the corresponding author upon reasonable request.

## Supplementary Materials

The following are available online at https://www.mdpi.com/article/10.3390/plants11040498/s1, Figure S1. (a) Profile of esterase isoenzyme of paulownia microshoots grown for one month on semisolid MS medium with 2 mg/L BAP and 0.1 mg/L NAA (lane BN); MS with 0.1 mg/L GA (lane G); MS with 2 mg/L BAP and 0.1 mg/GA (lane BG); or MS with 2 mg/L BAP, 0.1 mg/L NAA and 0.1 mg/L GA (lane BNG). (b) Diagrammatic shape illustrates the distribution of bands inside the esterase gel.; Figure S2. Two months age paulownia plant after acclimatization and transferring to open conditions; Figure S3. RAPD-PCR amplification patterns obtained from different primers of ten shoot lines of paulownia subjected for 14 successive subcultures on semisolid MS medium with 2 mg/L BAP and 0.1 mg/L NAA. Lane M: 50 bp DNA ladder.; Figure S4. ISSR-PCR amplification patterns obtained from different primers of ten shoot lines of paulownia subjected for 14 successive subcultures on semisolid MS medium with 2 mg/L BAP and 0.1 mg/L NAA. Lane M: 50 bp DNA ladder.; Figure S5. RAPD-PCR amplification patterns obtained from different primers of five selected-NaCl tolerant lines (lanes 2–6) in comparison to that of control (lane 1). M: 50 bp DNA ladder.; Figure S6. ISSR-PCR amplification patterns obtained from different primers of five selected-NaCl tolerant lines (lanes 2–6) in comparison to that of control (lane 1). M: 50 bp DNA ladder.
