# Supplementary material for: Interaction between Growth Regulators Controls In Vitro Shoot Multiplication in Paulownia and Selection of NaCl-Tolerant Variants
Source: Plants (Basel). 2022 Feb 11;11(4):498. doi: 10.3390/plants11040498 (PMC8878327; doi:10.3390/plants11040498)
Supplement: Supplementary file 1 [file plants-11-00498-s001.zip › plants-1532218-supplementary.pdf]

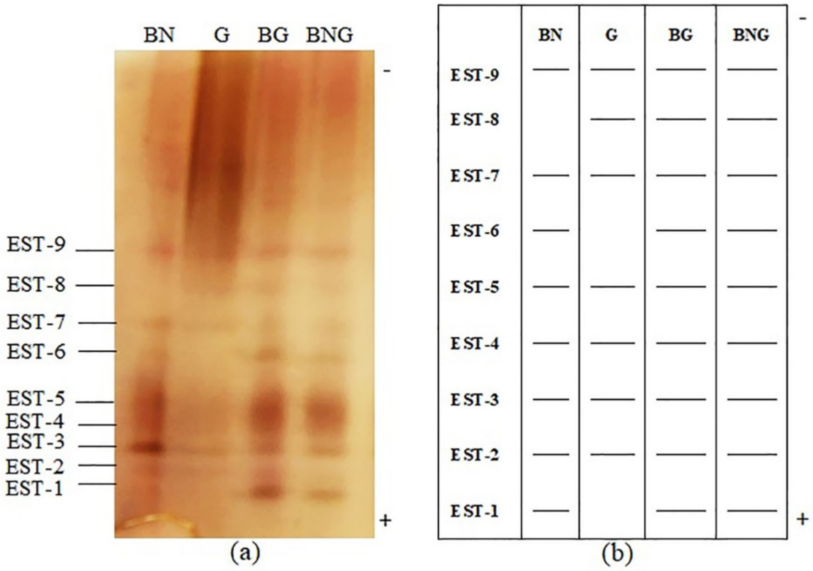

Figure S1. (**a**) Profile of esterase isoenzyme of paulownia microshoots grown for one month on semisolid MS medium with 2 mg/l BAP and 0.1 mg/l NAA (lane BN); MS with 0.1 mg/l GA (lane G); MS with 2 mg/l BAP and 0.1 mg/GA (lane BG); or MS with 2 mg/l BAP, 0.1 mg/l NAA and 0.1 mg/l GA (lane BNG). (**b**) Diagrammatic shape illustrates the distribution of bands inside the esterase gel.

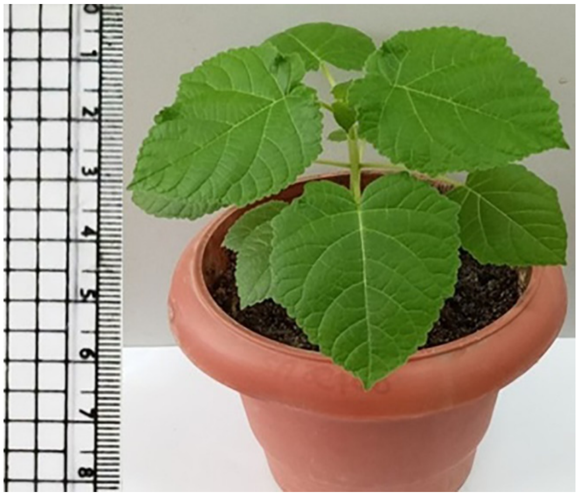

Figure S2. Two months age paulownia plant after acclimatization and transferring to open conditions

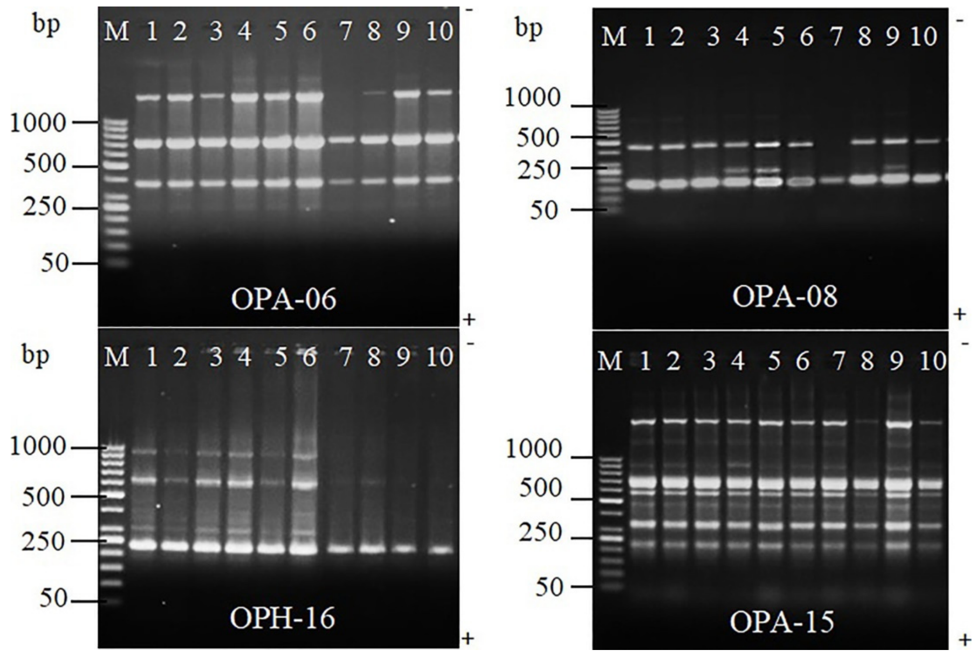

Figure S3. RAPD-PCR amplification patterns obtained from different primers of ten shoot lines of paulownia subjected for 14 successive subcultures on semisolid MS medium with 2 mg/l BAP and 0.1 mg/l NAA. Lane M: 50 bp DNA ladder.

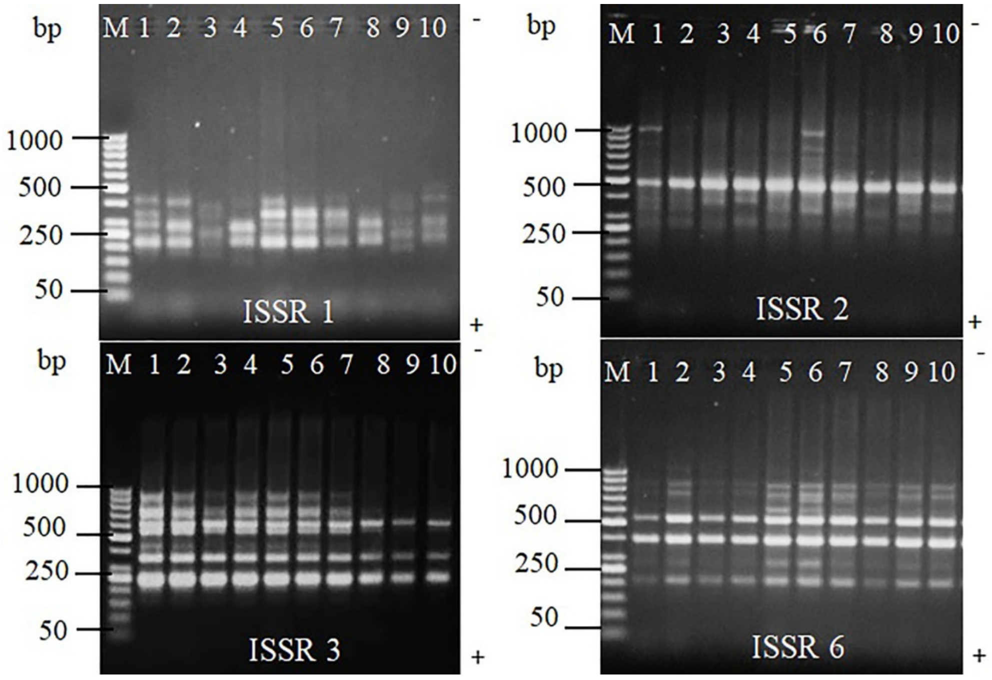

Figure S4. ISSR-PCR amplification patterns obtained from different primers of ten shoot lines of paulownia subjected for 14 successive subcultures on semisolid MS medium with 2 mg/l BAP and 0.1 mg/l NAA. Lane M: 50 bp DNA ladder.

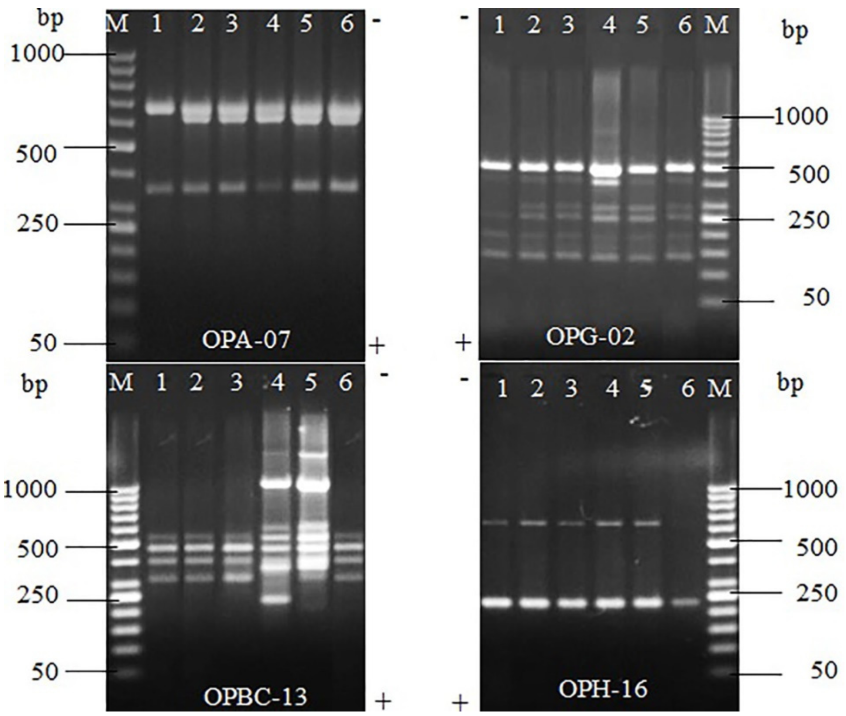

Figure S5. RAPD-PCR amplification patterns obtained from different primers of five selected-NaCl tolerant lines (lanes 2-6) in comparison to that of control (lane 1). M: 50 bp DNA ladder.

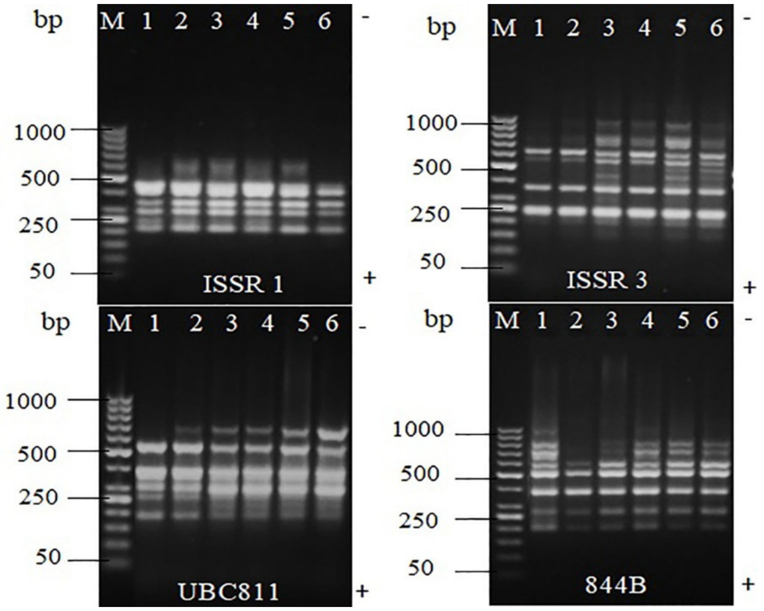

Figure S6. ISSR-PCR amplification patterns obtained from different primers of five selected-NaCl tolerant lines (lanes 2-6) in comparison to that of control (lane 1). M: 50 bp DNA ladder.
